# Curcumin ameliorates H _2_O _2_-induced injury through SIRT1-PERK-CHOP pathway in pancreatic beta cells: Curcumin alleviates ER stress

**DOI:** 10.3724/abbs.2022004

**Published:** 2022-02-16

**Authors:** Zhaohui Cao, Zhuan Wu, Tingting Duan, Cifei Tang, Di Huang, Xiaobo Hu

**Affiliations:** 1 Department of Biochemistry and Molecular Biology Hengyang Medical School University of South China Hengyang 421001 China; 2 Key Laboratory of Ecological Environment and Critical Human Diseases Prevention of Hunan Province Department of Education Hengyang Medical School University of South China Hengyang 421001 China; 3 Central Laboratory Guangxi Health Commission Key Laboratory of Glucose and Lipid Metabolism Disorders the Second Affiliated Hospital of Guilin Medical University Guilin 541000 China

**Keywords:** curcumin, ER stress, SIRT1, apoptosis

## Abstract

Oxidative stress and endoplasmic reticulum (ER) stress play crucial roles in pancreatic β cell destruction, leading to the development and progression of type 1 diabetes mellitus (T1DM). Curcumin, extracted from plant turmeric, possesses multiple bioactivities such as antioxidant, anti-inflammatory and anti-apoptosis properties *in vitro* and *in vivo*. However, it remains unknown whether curcumin improves ER stress to prevent β cells from apoptosis. In this study, we aim to investigate the role and mechanism of curcumin in ameliorating H _2_O _2_-induced injury in MIN6 (a mouse insulinoma cell line) cells. Cell viability is examined by CCK8 assay. Hoechst 33258 staining, TUNEL and flow cytometric assay are performed to detect cell apoptosis. The relative amounts of reactive oxygen species (ROS) are measured by DCFH-DA. WST-8 is used to determine the total superoxide dismutase (SOD) activity. Protein expressions are determined by western blot analysis and immunofluorescence staining. Pretreatment with curcumin prevents MIN6 cells from H _2_O _2_-induced cell apoptosis. Curcumin decreases ROS generation and inhibits protein kinase like ER kinase (PERK)-C/EBP homologous protein (CHOP) signaling axis, one of the critical branches of ER stress pathway. Moreover, incubation with curcumin activates silent information regulator 1 (SIRT1) expression and subsequently decreases the expression of CHOP. Additionally, EX527, a specific inhibitor of SIRT1, blocks the protective effect of curcumin on MIN6 cells exposed to H _2_O _2_. In sum, curcumin inhibits the PERK-CHOP pathway of ER stress mediated by SIRT1 and thus ameliorates H _2_O _2_-induced MIN6 cell apoptosis, suggesting that curcumin and SIRT1 may provide a potential therapeutic approach for T1DM.

## Introduction

Type 1 diabetes mellitus (T1DM) is a multifactorial disease with damaged pancreatic β cells, characterized by insulin deficiency and hyperglycemia [ 1, 2]. Despite many efforts, the current therapeutic options are not satisfactory in clinics, primarily because of side effects. Therefore, new therapeutic strategies aimed to protecting β cells from apoptosis or promoting β cell survival are continually being searched to better treat the disease.

Endoplasmic reticulum (ER) is an organelle in all eukaryotic cells, participating in protein synthesis, modification and calcium homeostasis. Pancreatic β cells are considered to be under high ER stress because a large amount of insulin is synthesized and processed in the ER [3]. In order to restore ER homeostasis, cells trigger the unfolded protein response (UPR). Accumulating data have demonstrated that severe ER stress and excessive UPR are related to pancreatic β-cell loss and diabetes [4]. Particularly, PKR‐like endoplasmic reticular kinase (PERK) has been identified as an important sensory element and major injury pathway initiation factor of ER stress in β-cell apoptosis and development of T1DM [ 5, 6]. The major regulators of the PERK signaling pathway include eukaryotic initiation factor 2α (eIF2α), activating transcription factor 4 (ATF4), and C/EBP homologous protein (CHOP). Hence, we focused on investigating the PERK pathway of ER stress in this study.

Curcumin, a well-known polyphenolic compound extracted from the rhizomes of turmeric *Curcuma* longa [7], has attracted attention with beneficial antidiabetic properties, partly due to its antioxidant, anti-inflammatory and glycohydrolase inhibitory effects [ 8– 10]. Furthermore, curcumin has been reported to directly affect pancreatic β cell survival by alleviating oxidative stress [ 11, 12]. As we known, oxidative stress is tightly associated with ER stress. Increased reactive oxygen species (ROS) can disturb calcium homeostasis, redox status and protein modification in the ER, resulting in ER stress. Conversely, ER stress increases ROS production and induces oxidative stress [13]. However, whether curcumin can protect pancreatic β cells through inhibiting ER stress-induced apoptosis is still ambiguous. Thus, we aim to explore and illustrate the underlying mechanisms.

Silent information regulator 1 (SIRT1) is a nicotinamide adenine dinucleotide (NAD)-dependent histone deacetylase, which plays essential roles in regulating various cellular biological processes, including stress resistance, inflammation, cell proliferation, cell apoptosis and cellular homeostasis [14]. Previous studies revealed the protective effect of SIRT1 on different cells under stress conditions [ 15, 16]. Furthermore, accumulating evidence supported that SIRT1 could increase insulin secretion and promote pancreatic β-cell survival [17]. Thus, we speculated that SIRT1 might be involved in the protective role of curcumin in MIN6 cells under stress conditions.

In the present study, we aim to investigate whether CUR protects MIN6 cells from stress injuries through inhibiting the PERK/CHOP pathway of ER stress mediated by SIRT1. The results of the present study provide clues for future investigation into the protective effects of curcumin at the molecular level, which is conducive to the treatment of diabetes.

## Materials and Methods

### Reagents

Curcumin, GSK2606414, Hoechst 33258, DAPI, 2′,7′-dichlorodihydrofluorescein diacetate (DCFH-DA), and EX527 were purchased from Sigma Aldrich (St Louis, USA). Dulbecco’s modified Eagle’s medium (DMEM) and fetal bovine serum (FBS) were purchased from Gemini Bio-Products (909-108; Woodland, USA). CCK8 reagent was obtained from Dojindo (Kumamoto, Japan). Annexin-V-FITC Apoptosis Detection kit was obtained from Keygen Biotech (KGA107; Nanjing, China). TUNEL Staining kit, WST-8 Assay kit, and RIPA buffer were purchased from Beyotime (Shanghai, China). BCA Protein assay kit was from Sangon Biotech (Shanghai, China).

### Cell culture

MIN6 cells were purchased from the Cell Bank of the Chinese Academy of Sciences (Shanghai, China), and cultured at 37°C in high glucose DMEM, containing 15% FBS, 100 U/mL penicillin and 100 μg/mL streptomycin with 5% CO _2_. Cells were used for experiments or subcultured when cells reached 80% confluence.

### Cell viability assay

The cytotoxicity of curcumin and H _2_O _2_ on MIN6 cells was measured by CCK8 assay. Briefly, cells were seeded in 96-well plates at 1.5×10 ^4^ cells/well and cultured for 12 h. Next, cells were treated with different concentrations of H _2_O _2_ for different time, with or without curcumin pretreatment for 1 h. Then, 10 μL CCK-8 solution was added per well, cultured for 2 h, and the optical density (OD) were measured at 450 nm. Cell viability was determined by comparing the number of viable cells to that of the control in which the viability was defined as 100%.

### Determination of intracellular ROS

Intracellular ROS concentrations were measured using DCFH-DA. Briefly, cells were seeded in 6-well plates at a density of 2×10 ^5^ cells/well. After treatment, the cells were washed with PBS thrice and then fixed with 4% paraformaldehyde (PFA) for 10 min. DCFH-DA (10 μM) was then added into each well and incubated in the dark for 30 min at 37°C. Cells were washed with PBS thrice, and ROS generation was observed under a fluorescence microscope (Olympus, Tokyo, Japan). ROS level was determined by comparing to that of control which was defined as 1.

### WST-8 assay

Total Superoxide Dismutase (SOD) activity was measured using the WST-8 Assay kit following the manufacturers’ instructions. Briefly, MIN6 cells were seeded in 6-well plates at a density of 2×10 ^5^ cells/well. After treatment, cells were incubated with the reaction solution for 30 min at 37°C according to the instructions of the kit. The absorbance was measured at a wavelength of 450 nm with a microplate reader.

### Flow cytometric analysis

Briefly, MIN6 cells were seeded in 6-well plates at 2×10 ^5^ cells/well and exposed to 100 μM H _2_O _2_ for 24 h, with or without 1 h of pretreatment with 20 μM curcumin. Cells were collected and double-stained with Annexin-V-FITC and propidium iodide (PI) using Annexin V-FITC Apoptosis Detection kit according to the manufacturer’s instruction. The apoptotic rate (%) in each group was measured and analyzed with the flow cytometer (BD, Piscataway, USA).

### Hoechst 33258 staining

MIN6 cells were seeded in a 6-well plate at a density of 2×10 ^5^ cells/well. After attachment, the cells of different groups were treated as described above. Subsequently, the cells were washed with PBS thrice and then fixed with 4% PFA for 10 min. Hoechst 33258 was then added into each well and incubated for 15 min at 37°C. After three times wash with PBS, the cells were observed under a fluorescence microscope (Olympus).

### DAPI staining

Briefly, MIN6 cells were fixed with 4% PFA for 10 min, washed with PBS, and incubated with DAPI. An Optika XDS-2 inverted microscope with an M-795 fluorescence system (Optika, Ponteranica, Italy) was used for imaging the nuclear fragmentation and condensation.

### TUNEL assay

TUNEL was used to detect the apoptotic levels in MIN6 cells after different treatments for 24 h. Briefly, MIN6 cells were fixed with 4% PFA for 30 min at room temperature and permeabilized for 5 min using 0.3% Triton X-100. Then cells were stained using a TUNEL Staining kit according to the manufacturer’s instructions. The slides were covered and incubated at 37°C for 60 min in the dark. Finally, TUNEL-positive cells were visualized by green fluorescence and images were captured under a fluorescence microscope.

### Western blot analysis

Protein was extracted from the cultured cells using RIPA buffer. A total of 30 μg proteins were separated by sodium dodecyl sulphate-polyacrylamide gel electrophoresis (SDS-PAGE). Then, proteins were transferred to polyvinylidene difluoride (PVDF) membranes. The membranes were blocked in TBST containing 5% skimmed milk, and incubated with the primary antibodies including: anti-GRP78 (1:1000; Sigma Aldrich), anti-PERK (1:1000; Sigma Aldrich), anti-p-PERK (1:500; Sigma Aldrich), anti-eIF2α (1:1000; Abcam, Cambridge, UK), anti-p-eIF2α (1:500; Abcam), anti-ATF4 (1:1000; Sigma Aldrich), anti-CHOP (1:500; Sigma Aldrich), anti-SIRT1 (1:1000; CST, Beverly, USA), anti-Bcl-2 (1:1000; Proteintech, Wuhan, China), Bax (1:1000; Proteintech), anti-Mcl-1(1:1000; Sigma), anti-caspase 3 (1:1000; CST), anti-caspase 9 (1:1000; CST), anti-cleaved caspase 3 (1:500; Sigma Aldrich), and anti-β-actin (1:1000; Origene, Rockville, USA), followed by incubation with the corresponding horseradish peroxidase (HRP)-conjugated secondary antibodies. Finally, chemiluminescent substrate (Beyotime) was used for signal visualization and detection. The density of each band was normalized to its respective loading control (β-actin). The immunoblots were quantified by densitometric analysis.

### Immunofluorescence staining

Immunofluorescence staining was performed using a standard protocol. Briefly, after three times wash with PBS, the cells were fixed with 4% PFA for 30 min at room temperature and permeabilized for 5 min using 0.3% Triton X-100. After being blocked with 5% bovine serum albumin in PBS for 2 h at 37°C, the cells were incubated with the primary antibody against p-eIF2α, ATF4 and SIRT1 overnight at 4°C. After being washed three times, the cells were incubated with Fluorescein (FITC)-AffiniPure conjugated second antibody for 60 min at room temperature. Cell nuclei were stained with DAPI for 10 min at room temperature. Fluorescence images were immediately captured under a fluorescence microscope (Olympus). p-eIF2α, ATF4 and SIRT1 levels were expressed as a percentage of the control.

### Statistical analysis

Data are presented as the mean±SEM from at least three experiments. Standard statistical software (SPSS for Windows, version 13.0; IBM, New York, USA) was used for data analysis. Statistical analysis was performed using unpaired Student’s *t* test, and *P*<0.05 was considered statistically significant.

## Results

### Effect of curcumin on H _2_O _2_-induced MIN6 cell viability

**Figure 1:**
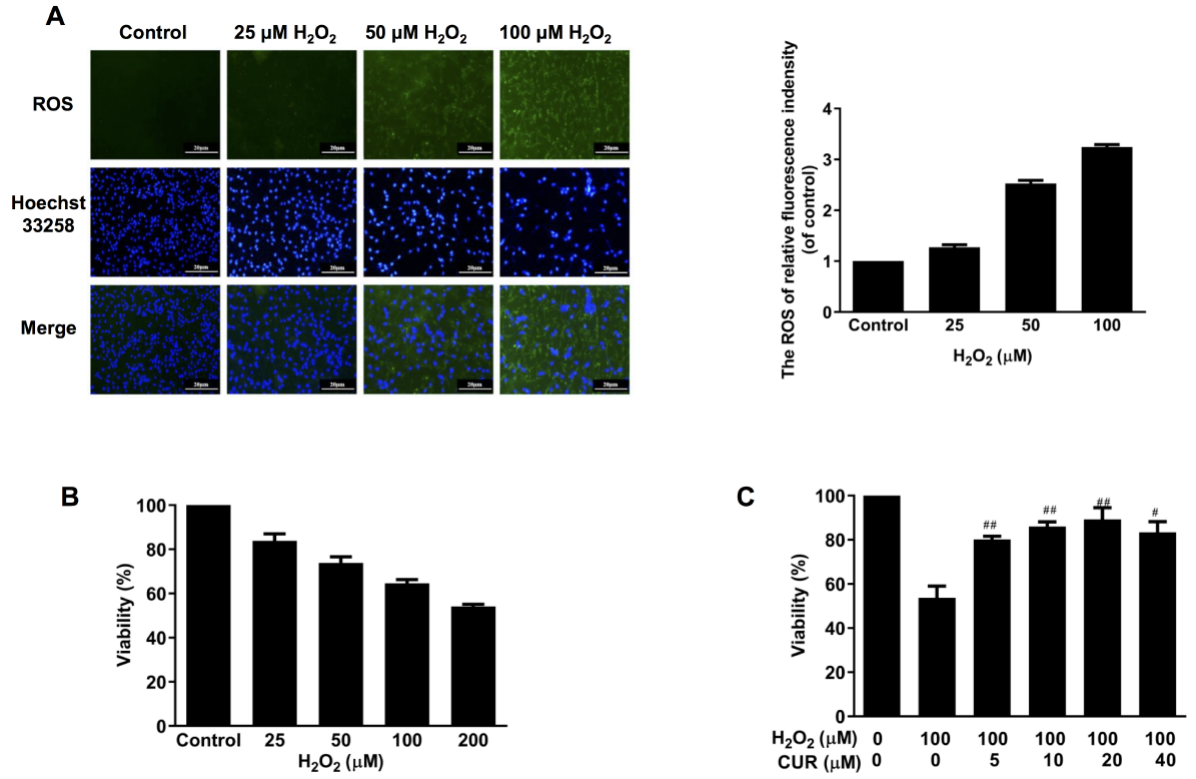
Figure1 Effects of curcumin on the viability of H _2_O _2_-induced MIN6 cells (A) MIN6 cells were treated with various concentrations of H2O2 for 24 h. ROS production was detected by DCFH-DA fluorescent probe assay. Nuclear changes in the cells were assessed by Hoechst 33258 staining (scar bar=20 μm). (B) CCK8 assay was performed to measure the cytotoxic effects of various concentrations of H2O2 on MIN6 cells after 24 h of incubation. (C) Cell viability of H2O2-treated MIN6 cells with various concentration of curcumin pretreatment for 1 h was measured. Data are presented as the mean±SEM (n=4). #P<0.05, ##P<0.01, vs H2O2 group. Con: control; CUR: curcumin.

To mimic pancreatic β-cell stress condition *in vivo*, H _2_O _2_ was used in the present study. Firstly, MIN6 cells were incubated with various concentrations of H _2_O _2_ (0, 25, 50, and 100 μM) for 24 h. ROS production was detected using DCFH-DA fluorescent probe. Figure 1A showed that when MIN6 cells were exposed to different concentrations of H _2_O _2_ for 24 h, ROS production was markedly increased. MIN6 cells that were incubated with H _2_O _2_ exhibited a typical apoptotic morphological change, including condensed chromatin in apoptotic nuclear and the formation of apoptotic bodies ( Figure 1A). Based on our previous study [18], 24 h of treatment with 100 μM H _2_O _2_ is the optimal condition to construct stress injury model of MIN6 cells because more than 60% of the cells remained viable ( Figure 1B). Next, cells were treated with 100 μM H _2_O _2_, with 1 h of pretreatment with different concentrations (5, 10, 20, and 40 μM) of curcumin to validate the protective effect of curcumin on H _2_O _2_-induced cell death. As shown in Figure 1C, 20 μM of curcumin exhibited better efficacy in preventing MIN6 cells from apoptosis, with about 85% of the cells remained viable. Therefore, 20 μM of curcumin was chosen to investigate the protective effect on 100 μM H _2_O _2_-induced cytotoxicity in the subsequent experiments.

### Curcumin prevents MIN6 cells from H _2_O _2_-induced apoptosis

**Figure 2:**
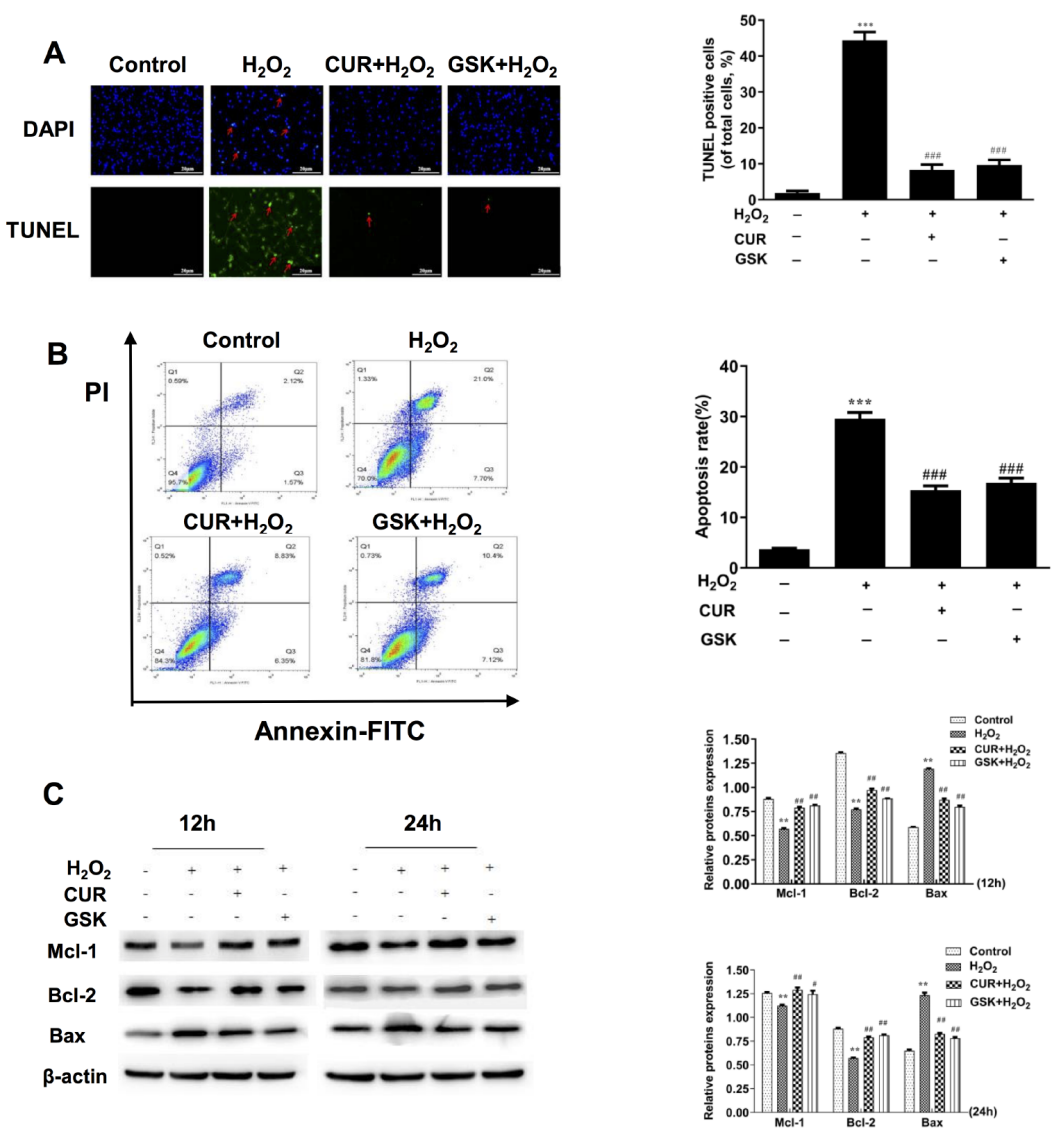
Figure2 Curcumin protects MIN6 cells against H _2_O _2_-induced apoptosis MIN6 cells were treated with H2O2 for 24 h with curcumin (20 μM) or GSK2606414 (1 μM) pretreatment for 1 h. (A) Nuclear changes of the cells were detected by DAPI staining (scale bar=20 μm). Apoptotic MIN6 cells were examined using TUNEL fluorescence immunocytochemistry in green (scale bar=20 μm). (B) Apoptotic MIN6 cell percentage was determined by flow cytometry. (C) MIN6 cells were treated with H2O2 for 12 or 24 h with curcumin (20 μM) or GSK2606414 (1 μM) pretreatment for 1 h. The protein levels of Bcl-2, Mcl-1 and Bax in each group were detected by western blot analysis. β-Actin was used as an internal control. All the values are presented as the mean±SEM (n=3). **P<0.01, ***P<0.001 vs control group. #P<0.05, ##P<0.01, ###P<0.001 vs H2O2 group. Con: control; CUR: curcumin; GSK: GSK2606414.

Our previous study verified that MIN6 cell death induced by 100 μM H _2_O _2_ was resulted from cell apoptosis rather than necrosis [18]. Hereby, we investigated whether curcumin plays a cytoprotective role in MIN6 cells through inhibiting cell apoptosis. As shown in Figure 2A, MIN6 cells treated with H _2_O _2_ underwent an obvious apoptotic morphological change, including chromatin condensation and the formation of apoptotic bodies, while cells pretreated with 20 μM curcumin showed almost normal nuclear material with evenly distributed chromatin as revealed by DAPI staining. Consistent with the results of DAPI, few TUNEL-positive apoptotic cells were observed in the control group, while a number of apoptotic cells were observed in H _2_O _2_-treated cells (44.3%; *P*<0.001). In addition, pretreatment with curcumin significantly decreased MIN6 cells apoptosis as demonstrated by the low apoptotic rate in the H _2_O _2_+20 μM CUR group (8.3%; *P*<0.001). Similarly, quantification of apoptotic MIN6 cells was performed by flow cytometry. As shown in Figure 2B, with exposure to H _2_O _2_ alone for 24 h, the percentage of apoptotic cells was increased to 28.7%, whereas pretreatment with 20 μM curcumin markedly reduced the apoptotic rates to 15.2%.

To further confirm the above results, apoptosis-related proteins were detected by western blot analysis. Compared with those in the H _2_O _2_ group at 12 and 24 h, curcumin pretreatment significantly decreased pro-apoptotic protein (Bax) expression and increased anti-apoptotic protein (Bcl-2 and Mcl-1) expression, in MIN6 cells ( Figure 2C). Taken together, these data confirmed that curcumin has the protective effect against H _2_O _2_-induced apoptosis in MIN6 cells.

### Curcumin alleviates H _2_O _2_-induced oxidative stress in MIN6 cells

**Figure 3:**
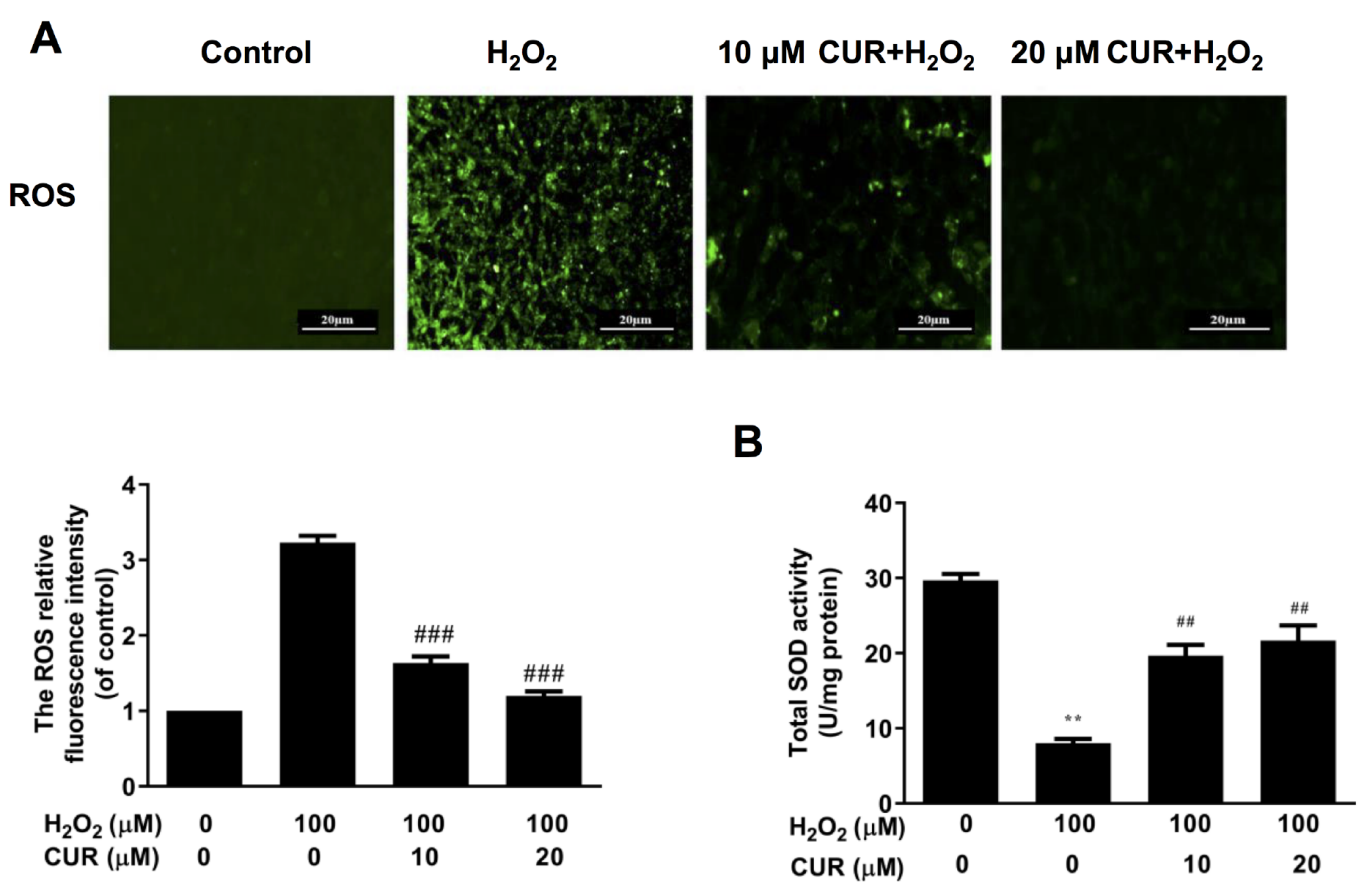
Figure3 Curcumin alleviates H _2_O _2_-induced oxidative stress in MIN6 cells MIN6 cells were treated with H2O2 for 24 h with curcumin (0, 10, and 20 μM) pretreatment for 1 h. (A) ROS levels were detected by DCFH-DA assay. (B) T-SOD activity was measured by WST-8 assay. All the values are presented as the mean±SEM (n=3). **P<0.01, vs control group. ##P<0.01, vs H2O2 group. Con: control; CUR: curcumin.

Many of the beneficial effects of curcumin are resulted from its antioxidant properties. Thus, we investigated whether curcumin could inhibit H _2_O _2_-induced oxidative stress in MIN6 cells. As shown in Figure 3A, the production of intracellular ROS was elevated after exposure to 100 μM H _2_O _2_ alone for 24 h, while pre-treatment with 10 μM or 20 μM curcumin markedly reduced the intracellular ROS level. Intracellular superoxide dismutase (SOD, an antioxidant enzyme) level was assessed to evaluate the protective effect of curcumin on H _2_O _2_-induced oxidative damage. Figure 3B showed that H _2_O _2_ caused a significant decrease of SOD level, whereas pretreatment with 10 μM or 20 μM curcumin obviously increased H _2_O _2_-induced SOD level ( *P*<0.01). These data clearly indicated that curcumin could alleviate H _2_O _2_-induced oxidative stress in MIN6 cells.

### Curcumin attenuates H _2_O _2_-induced MIN6 cell apoptosis by inhibiting PERK-eIF2α-ATF4-CHOP pathway

**Figure 4:**
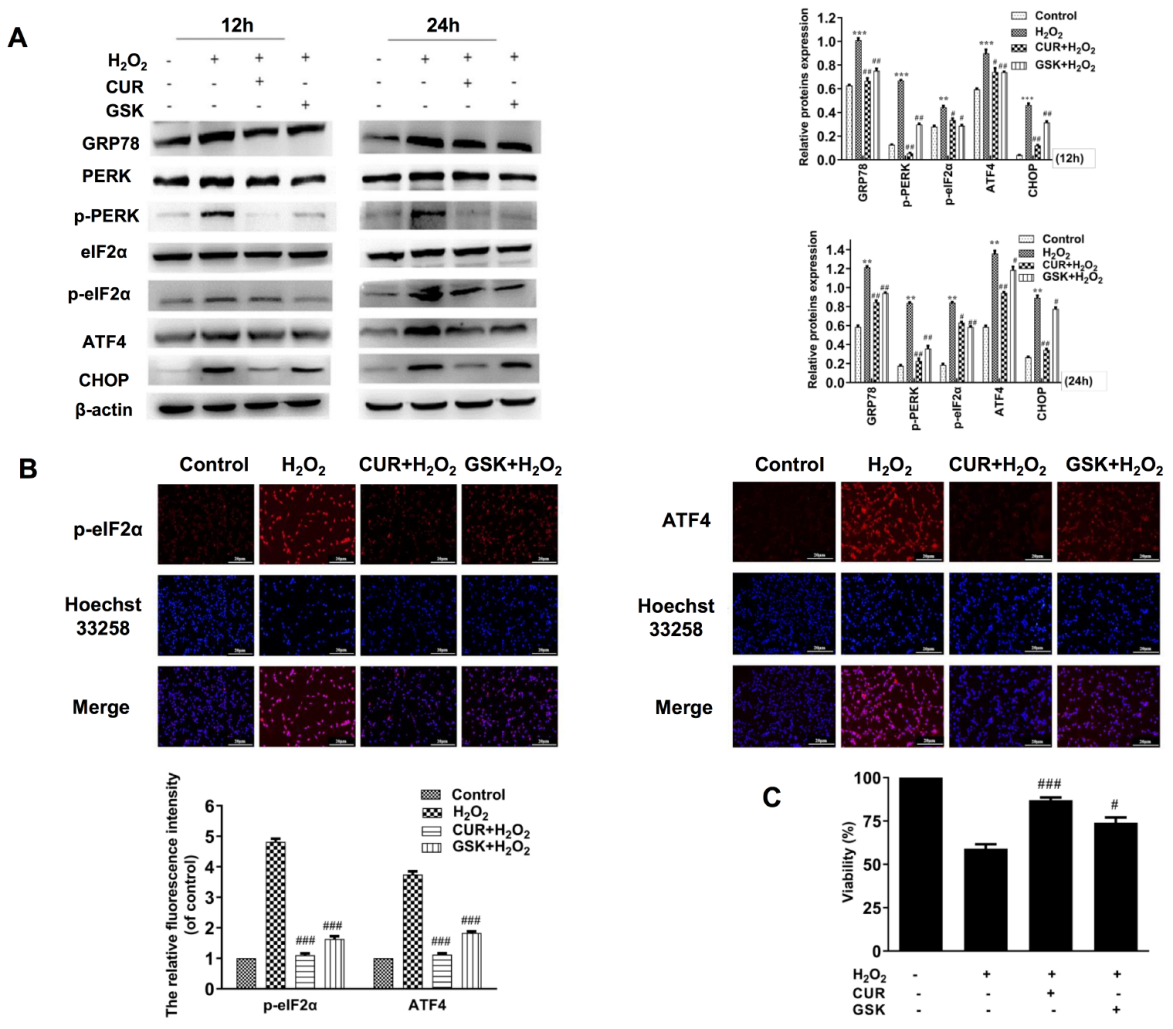
Figure4 Curcumin attenuates H _2_O _2_-induced MIN6 apoptosis by inhibiting PERK-eIF2α-ATF4-CHOP pathway (A) MIN6 cells were treated with H2O2 for 12 or 24 h with 20 μM curcumin or 1 μM GSK pretreatment for 1 h. The key molecules of ER stress pathways in each group were detected by western blot analysis. β-Actin was used as an internal control. (B) MIN6 cells were treated with H2O2 for 24 h with 20 μM curcumin or 1 μM GSK pretreatment for 1 h. p-eIF2α and ATF4 were measured by immunofluorescence staining assay. (C) Cell viability was detected by CCK-8 assay. **P<0.01, ***P<0.001 vs control group. #P<0.05, ##P<0.01, ###P<0.001 vs H2O2 group. con: Control; CUR: curcumin; GSK: GSK2606414.

Both oxidative stress and ER stress are associated with the H _2_O _2_-induced MIN6 apoptosis [19]. Therefore, we examined the levels of biomarkers in the PERK pathway by western blot analysis and immunofluorescence staining analysis. As shown in Figure 4A, GRP78, p-PERK, p-eIF2α, ATF4, and CHOP levels were significantly increased at 12 h and lasted until 24 h after challenging with H _2_O _2_, which was partially reversed by 20 μM curcumin pretreatment. The total PERK and eIF-2α remained unchanged compared to the control group ( Figure 4A). The data of immunofluorescence staining of p-eIF2α and ATF4 are consistent with the results of western blot analysis ( Figure 4B). Therefore, curcumin attenuates H _2_O _2_-stimulated apoptosis probably by suppressing PERK-CHOP axis in MIN6 cells.

To further confirm the above results, GSK2606414, a specific inhibitor of PERK, was used to inhibit ER stress. MIN6 cells were exposed to 1 μM GSK2606414 for 1 h before H _2_O _2_ treatment. As expected, GSK2606414 dramatically reduced the levels of p-PERK, p-eIF2α, ATF4 and CHOP in MIN6 cells compared with those in the H _2_O _2_ group at 12 or 24 h ( Figure 4A,B). Furthermore, CCK8 results showed that GSK2606414 could restore MIN6 cell viability ( Figure 4C). Flow cytometry and TUNEL staining were also applied to examine the apoptosis rate of MIN6 cells. As shown in Figure 2A,B, pretreatment with GSK2606414 effectively decreased the apoptotic rate compared with that in the H _2_O _2_ group. Taken together, all these results fully confirmed that curcumin prevents H _2_O _2_-treated MIN6 from apoptosis by inhibiting PERK-CHOP pathway of ER stress.

### Curcumin blocks PERK-eIF2α-ATF4-CHOP axis of the ER stress via increasing SIRT1 expression in H _2_O _2_-treated MIN6 cells

**Figure 5:**
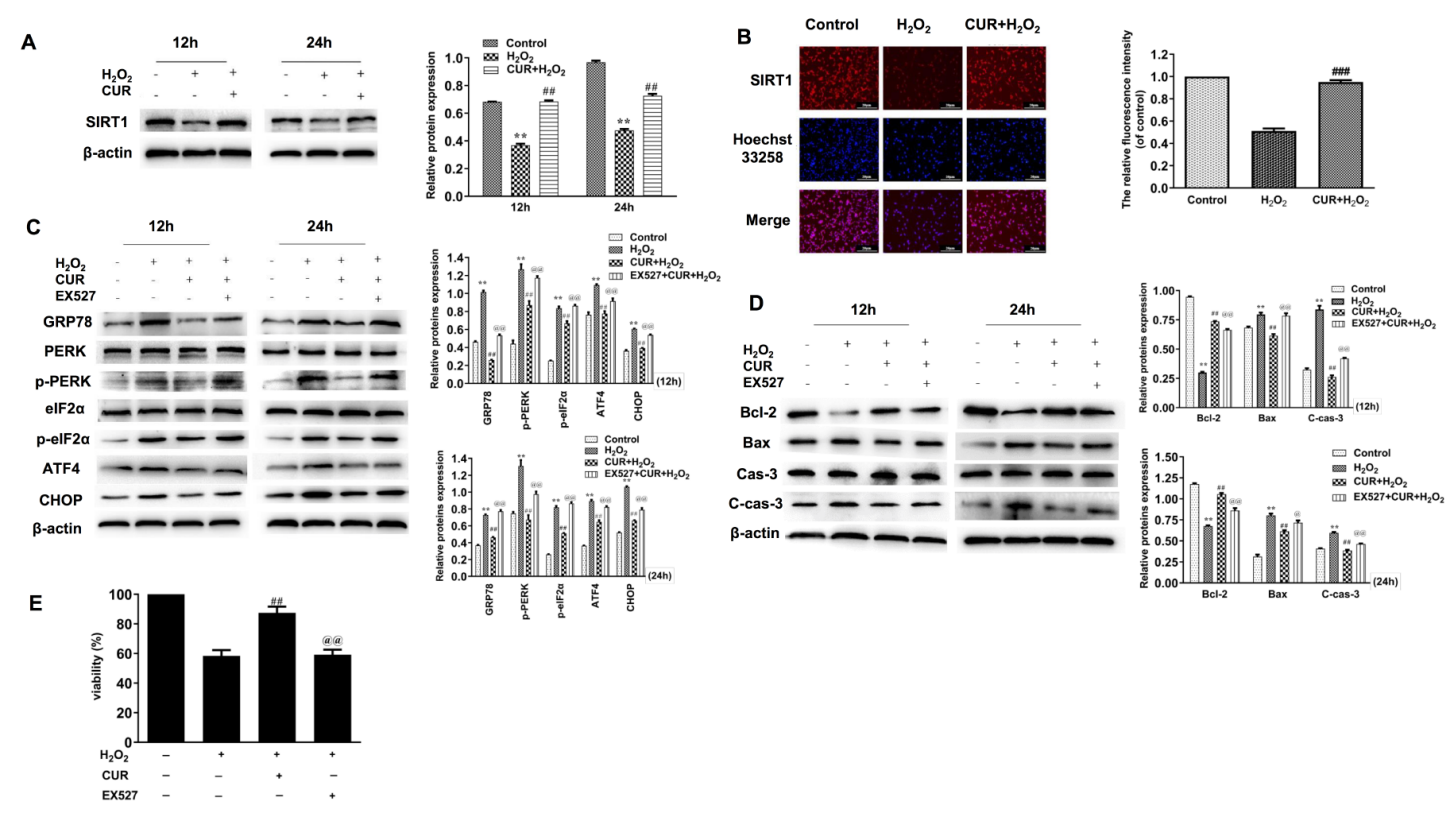
Figure5 Curcumin blocks ER stress via increasing SIRT1 expression in H _2_O _2_-treated MIN6 cells (A) MIN6 cells were treated with H2O2 for 12 or 24 h with or without 20 μM curcumin pretreatment for 1 h. SIRT1 was detected by western blot analysis. β-Actin was used as an internal control. (B) SIRT1 was measured by immunofluorescence staining assay. (C) MIN6 cells were treated with H2O2 for 12 or 24 h with 20 μM curcumin or 40 μM EX527 pretreatment for 1 h. The key molecules of ER stress pathways were detected by western blot analysis. β-Actin was used as an internal control. (D) Bax, Bcl-2, and caspase3/cleaved caspase 3 levels were detected by western blot analysis. (E) Cell viability was detected by CCK-8 assay. All the values are presented as the mean±SEM (n=3). **P<0.01, vs control group. ##P<0.01, ###P<0.001, vs H2O2 group. @P<0.05, @@P<0.01, vs CUR+H2O2 group. Con: control; CUR: curcumin.

To determine whether SIRT1 is involved in H _2_O _2_-induced apoptosis in MIN6 cells, we firstly examined the expression of SIRT1 in response to H _2_O _2_ treatment. As shown in Figure 5A, H _2_O _2_ decreased the expression of SIRT1 in MIN6 cells, while pretreatment with curcumin abrogated the effect of H _2_O _2_. Similarly, the data of immunofluorescence staining are consistent with the results of western blot analysis ( Figure 5B). To further evaluate the role of SIRT1 in the effect of curcumin on alleviating ER stress in MIN6 cells exposed to H _2_O _2_, we treated cells with EX527, a known SIRT1-specific inhibitor. As shown in Figure 5C, 40 μM EX527 markedly inhibited the protective role of curcumin in downregulating the expressions of p-PERK, p-eIF2α, ATF4 and CHOP, compared with those in the H _2_O _2_+CUR group at 12 and 24 h. These data proved that SIRT1 is involved in the PERK-CHOP signal pathway in MIN6 cell apoptosis, indicating that curcumin-alleviated ER stress is mediated by increased SIRT1 expression. To further verify the role of SIRT1 in β-cell survival, the expressions of apoptosis-related proteins (Bax and Bcl-2) and caspase 3/cleaved caspase 3 were also measured, and cell viability of MIN6 cells was detected. As shown in Figure 5D,E, EX527 blocked the protective role of curcumin in MIN6 cells apoptosis, including decreased Bcl-2 expression, increased Bax and promoted caspase 3 activation ( Figure 5D), and ultimately decreased cell viability ( Figure 5E). Collectively, our data cumulatively indicated that SIRT1 is essential for the protective effect of curcumin in ER stress-induced MIN6 cell apoptosis.

## Discussion

Increasing evidence indicates that pancreatic β-cells are highly fragile and vulnerable to cell stress factors, thus excessive oxidative stress and ER stress contribute to pancreatic β-cell failure and loss [20]. As common ROS, H _2_O _2_ was reported to freely diffuse through the pancreatic β-cell membrane, depending on a transmembrane protein called peroxiporins [21]. Since alterations in redox status directly or indirectly affect ER homeostasis and protein folding, the production of ROS immediately initiates ER stress. If the UPR-mediated efforts fail to restore ER homeostasis, apoptosis is activated. Therefore, we exposed MIN6 cells to H _2_O _2_ to mimic a damage model *in vivo*, for subsequent investigation. Our results demonstrated that treatment of MIN6 cells with 100 μM H _2_O _2_ induced chromatin margination and nuclear condensation, increased ROS production, decreased cell viability, and promoted MIN6 cells apoptosis ( Figure 1). Then, we employed multidisciplinary approaches to study the beneficial role of curcumin on MIN6 cell viability and apoptosis. We found that curcumin pretreatment significantly increased MIN6 viability, decreased MIN6 apoptotic rate (Figures 1, 2), and also markedly reduced ROS production and promoted T-SOD activity ( Figure 3). Thus, the next question is the potential mechanism by which curcumin exerts its protective role in MIN6 cell overloaded with H _2_O _2_.

Recent studies revealed that curcumin alleviates the development and progression of various diseases by inhibition of ER stress. Feng et al. [22] demonstrated that curcumin inhibited chondrocytes apoptosis and ameliorated osteoarthritis progression in a rat model through the PERK-eIF2α-CHOP pathway. Yu *et al*. [23] found that curcumin attenuated angiotensin II-induced podocyte injury and apoptosis by inhibiting ER stress. In addition, curcumin was reported to effectively improve pancreatic β cell function and prevent β cell apoptosis in streptozotocin (STZ)-induced T1DM rat model [11]. Similarly, evidence showed that curcumin ameliorated the heart and kidney damages in STZ-induced T1DM mice model [24]. Thus, we hypothesized that the protective role of curcumin in H _2_O _2_-treated MIN6 cells might be associated with inhibition of ER stress.

Studies have confirmed that PERK-CHOP axis of ER stress is a key factor in pancreatic β cell damage and T1DM development *in vitro* and *in vivo* [ 5, 25]. CHOP can upregulate apoptotic protein (Bax) expression and downregulate anti-apoptotic protein (Bcl-2) expression, thus initiating cell apoptosis [ 18, 26]. In this study, we found that treatment with 100 μM H _2_O _2_ increased the phosphorylation of PERK and eIF2α, subsequently led to increased expressions of ATF4 and CHOP, indicating that ER stress is involved in MIN6 cell apoptosis induced by H _2_O _2_, which is consistent with the previous publication [18]. We further found that curcumin markedly counteracted H _2_O _2_-induced PERK and eIF2α phosphorylation, as well as ATF4 and CHOP expression. Furthermore, GSK2606414, a selective PERK inhibitor, was used as a positive control to confirm the above results. As illustrated in Figure 4, pretreatment with GSK2606414 also decreased CHOP expression and restored cell viability. Thus, these results demonstrated that curcumin plays a protective role in MIN6 cells through inhibiting ER stress.

Histone acetylation, as a post-translational modification, is involved in key cellular processes relevant to physiology and disease, such as gene transcription, signal transduction, protein folding and metabolism [27]. SIRT1, identified as an NAD-dependent deacetylase, functions in the cellular response to inflammatory, metabolic, and oxidative stresses [ 14– 16]. Since then, SIRT1 has been intensively studied because of its great potential for human health benefits. It was reported that SIRT1 regulates forkhead box O1 (FoxO1) translocation, thereby directing an anti-apoptotic transcriptional program that protects INS 832/13 cells (a rat insulinoma cell line) from nitric oxide-induced apoptosis [28]. Additionally, Artesunate protects pancreatic β-cells against cytokine-induced damage via inhibition of NF-κB activation by SIRT1 [29]. Resveratrol, another natural compound, has been extensively studied with its antioxidant, anti-inflammatory and anti-diabetic properties, while most of the beneficial effects are resulted from the activation of SIRT1 [30]. Since SIRT1 plays an important role in protecting pancreatic β-cells and alleviating T1DM progression, in this study we also explored whether the function of curcumin in inhibiting ER stress is mediated by SIRT1 expression. Our data showed that H _2_O _2_ decreased SIRT1 expression, which was reversed by pretreatment with 20 μM curcumin. To further confirm that curcumin inhibits ER stress via mediating the expression of SIRT1, EX527, a specific inhibitor of SIRT1, was used in this study. EX527 increased the expressions of ATF4 and CHOP, and also increased the ratio of p-PERK/PERK and p-eIF2α/eIF2α compared with those in the H _2_O _2_+CUR group, which partially abolished the protective effect of curcumin on ER stress in H _2_O _2_-treated MIN6 cells ( Figure 5). We previously found that 1,25-(OH) _2_D _3_ protects against H _2_O _2_-induced MIN6 cell apoptosis through inhibiting ER stress [18]. However, in this study we demonstrated that curcumin inhibits the PERK-CHOP pathway of ER stress, which is mediated by SIRT1.

Insulin secreted from pancreatic β-cells plays a critical role in maintaining blood glucose level in a normal physiological range. On one hand, human pluripotent stem cells-derived pancreatic β-like cell transplantation is an effective method for treating T1DM [31]. On the other hand, protection of pancreatic β-cells from apoptosis is considered as a new therapeutic strategy. In this study, SIRT1, as a positive regulator of MIN6 cell viability, potentially represents a new therapeutic target for the treatment of T1DM. Furthermore, curcumin increases SIRT1 expression in MIN6 cells under ER stress. In summary, curcumin exerts a protective effect on H _2_O _2_-induced MIN6 cell apoptosis via the inhibition of PERK-eIF2α-ATF4-CHOP signaling pathway of ER stress, which is mediated by SIRT1. The beneficial effects of curcumin and SIRT1 on pancreatic β-cells may provide a potential therapeutic approach for T1DM.
